# Assessment of depression and suicidal thoughts among female trail runners

**DOI:** 10.3389/fpsyg.2026.1753949

**Published:** 2026-08-11

**Authors:** Carolyn Hill, Cécile Vigne, Patrick Basset, David Baud, Volker Scheer

**Affiliations:** 1 Materno-Fetal and Obstetric Research Unit, Obstetric Service, Woman-Mother-Child Department, University Hospital of Lausanne (CHUV), University of Lausanne (UNIL), Lausanne, Switzerland; 2 Ultra Sports Science Foundation, Pierre-Bénite, France

**Keywords:** athlete psychology, depression, endurance sport, female athletes, mental health, PHQ-9, suicidality, trail running

## Abstract

This cross-sectional study aims to screen for depression and suicidal thoughts, as well as identify associated factors, among female trail runners. An online survey was used, collecting participants’ sociodemographic data, trail running habits, medical indicators, and screening for depression (using the PHQ-9) and suicidal thoughts (using Q9 of the PHQ-9). A total of 318 female trail runners answered the survey. Depression was defined as PHQ-9 ≥ 10, and suicidal ideation was assessed using item 9 of the PHQ-9. Statistical analyses included chi-square tests and multivariate logistic regression to identify factors associated with a positive screening for depression and suicidal thoughts. A total of 18.9% participants had a positive PHQ-9 score, screening positive for major depression. Factors associated with a positive screening for depression were unemployment (*p* = 0.004), younger age groups (*p* = 0.033), trail running fewer than 3 years (*p* = 0.002) and never having been pregnant or given birth (*p* = 0.013). Unemployment was the only independent risk factor for depression (*p* = 0.005). More than 3 years of trail running (*p* = 0.013) and parity ≥ 1 (*p* = 0.036) were independent protective factors for depression. Overall, 7.9% participants answered “yes” to question 9 (Q9) of the PHQ-9, indicating the presence of suicidal ideations. Factors associated with a positive screening for suicidal thoughts were weekly training distance >50 km (*p* = 0.020), previous diagnosis of an eating disorder (*p* < 0.001) and never having been pregnant (*p* = 0.008) or given birth (*p* = 0.033). We present novel data on depression and suicidal thoughts in female trail runners. Nearly 1 in 5 female trail runners screened positive for depression and nearly 1 in 13 screened positive for suicidal thoughts. This highlights the importance of creating awareness for mental health and providing education and access to appropriate support.

## Introduction

Depression affects about 5.7% of the general adult population and increases to 6.9% among women [World Health Organization (WHO), 2025]. It is a leading cause of disability around the world and largely contributes to the global burden of disease. Indeed, depressive disorders are associated with significant functional impairment, reduced quality of life, and increased mortality, particularly through suicide [World Health Organization (WHO), 2017]. Suicide is a leading cause of premature death, accounting for more than 1.1% of deaths worldwide in 2021 (World Health Organization, 2022). Numerous risk factors have been identified, most notably mental disorders, especially depression (Favril et al., 2022), as well as sociodemographic factors, including female sex (Ivey-Stephenson et al., 2022).

In recent years, mental health has gained increasing attention in sport, where athletes face unique physical and psychological demands. Although participation in sport is often considered protective due to its positive effects on physical and psychological well-being (Eather et al., 2023), evidence suggests that athletes remain vulnerable to mental health challenges (Gouttebarge et al., 2019). Studies have reported a higher prevalence of depression among elite athletes compared to the general population (Gouttebarge et al., 2019; Purcell et al., 2020). As a result, screening tools such as the Patient Health Questionnaire-9 (PHQ-9) have been used to assess depression in athletes. The PHQ-9 is a validated tool used to screen for major depression in the general population. It is included in the International Olympic Committee’s Sport Mental Health Assessment Tool 1 and has demonstrated strong validity in athlete populations (Taylor et al., 2023; Anderson et al., 2023). However, a positive screening on the PHQ-9 should be confirmed with further psychological assessment to confirm the diagnosis (Costantini et al., 2021).

Using the PHQ-9, a recent study, found that 11.3% of endurance runners screened positive for major depression, with female runners more affected than male (Scheer et al., 2025). The study also identified additional risk factors, including being under 28 years old, having lost training time to injury, more frequent weekly workouts and a previous self-reported history of depression (Scheer et al., 2025). Another study, employing a different depression screening questionnaire, reported a high prevalence (21.9%) of depressive symptoms among ultra-distance endurance runners, with female runners reporting nearly twice the rate of depression compared to males (Niering et al., 2024). Consistent with these findings, a meta-analysis reported that the prevalence of depressive symptoms in ultra-endurance runners ranged from 12.8 to 18.6% indicating that women athletes were most vulnerable to developing mental health issues (Thuany et al., 2023). Moreover, endurance and ultra-endurance runners report high rates of anxiety and sleep problems, both closely related to depression (Jacobson and Newman, 2017; Freeman et al., 2020). Associated factors were younger age, years participating in the sport, number of competitions per year and injury related absences (Scheer et al., 2025). In an additional study, the prevalence of suicidal thoughts was two times higher in endurance and ultra-endurance runners compared to the general population (Scheer et al., 2025). They also found that major risk factors for suicidal thoughts were previous diagnosis of depression, anxiety, being under 30 years old and competing in fewer competitions per year (Scheer et al., 2025).

Trail running is defined as off-road running in a natural environment with a maximum of 20% of paved roads on the total race course with distances ranging from a few kilometers to more than one hundred kilometers (Scheer et al., 2020; International Trail Running Association, 2025). This sport has grown rapidly in popularity worldwide, with, notably, female participation growing from 13% in 1997 to 46% in 2022 (Andersen, 2022). This discipline exposes participants to prolonged physical effort, challenging environmental conditions and demanding training sessions, factors that may increase susceptibility to mental health concerns. Despite these potential risks, mental health in trail running remains understudied, and data specific to women are scarce.

Given that female sex is a recognized risk factor for depression among endurance runners and that few studies have yet focused on trail running, evaluating mental health in female trail runners is essential to better understand their specific vulnerabilities and needs. Understanding the prevalence of depression and suicidal ideation in this population along with associated risk and protective factors, will help guide interventions, strengthen support strategies and ultimately promote athlete well-being. This study therefore aimed to screen for depression in female trail runners and identify potential factors associated to depressive symptoms and suicidal thoughts, in order to better inform athletes and their teams.

## Materials and methods

This study was conducted as a cross-sectional study among female trail runners. The participants filled out an online survey and the inclusion criteria were to be female, at least 18 years old and to regularly practice trail running (training at least once a week or competing). As part of the survey, informed consent was obtained from all participants. The study was approved by the Comité Ético Investigacíon from University Hospital Elche, Spain, and was conducted in accordance with the Declaration of Helsinki (World Medical Association, 2013).

The survey was administered via Google Forms and was available in four languages: English, French, Spanish and Portuguese. As described in a previous publication (Scheer et al., 2025), the link to the survey was available via the Ultra Sports Science Foundation’s website and remained active between October 2024 and March 2025. Participants were recruited through flyers and online links shared across social media, participating race organizers, and running related platforms (e.g., Ultra Trail Mount Blanc [UTMB®, Chamonix, France], UTMB® World Series, Patagonia Run, Ribera Salud, local running platforms, etc.). All data was self-reported and all questions had to be completed before moving on to the next one.

The questionnaire included information on participant’s characteristics (age, sex, height, weight, nationality, smoking, civil status, employment status), trail running habits (years training, running distance, weekly training kilometers and hours, performance level, yearly race participation) and medical indicators (menopause, pregnancies, births, contraception, libido, urinary incontinence, pelvic prolapse, stress fractures, eating disorder diagnosis). Distances categories were defined as short trail (<42 km), long trail (42-80 km) to ultra trail (>80 km). Performance level included self-classification of elite and non-elite trail runner, with elite runners defined as professional, or collegiate, participating at the national or international level (Gouttebarge et al., 2021).

The survey also contained the Patient Health Questionnaire-9 (PHQ-9; Kroenke et al., 2001; Keenan et al., 2023), a validated diagnostic screening tool, used to assess the risk of depression among the participants. This brief questionnaire measures the severity of depressive symptoms and suicidal thoughts over the preceding 14 days. It consists of 9 items each scored on a four-point Likert-scale for frequency of symptoms (0, not at all, 1, several days, 2, more than half the days, 3, nearly every day). The total score ranges between 0 to 27, with higher scores reflecting greater severity of depression and scores ≥ 10 identifying major depression. Additionally, the ninth item of the PHQ-9 (Q9:"Thoughts that you would be better off dead or of hurting yourself in some way”) was used as a surrogate for suicidal thoughts and risk (Kroenke et al., 2001). It has been shown to be a robust predictor of suicidal attempt and death regardless of age (Rossom et al., 2017).

In order to facilitate statistical modeling, continuous variables were categorized into ordinal groups, with cutoff points defined to ensure approximately equal numbers of participants in each category. The PHQ-9 scores were dichotomized into positive and negative categories based on the validated cutoff value. Responses to Q9 of the PHQ-9 were also dichotomized to indicate the presence or absence of suicidal thoughts. Differences in sociodemographic characteristics, trail running habits, and general health variables between runners with positive versus negative questionnaire scores, and between those with a positive and negative screening for suicidal thoughts, were assessed using the Pearson χ^2^ test for categorical variables. Multi variate logistic regressions were conducted to identify factors independently associated with a positive score for the PHQ-9 and odds ratios were used to examine these associations. This analysis was not conducted for Q9 due to the small number of positive cases (*n* = 25). All analyses were considered statistically significant at *p* < 0.05. Statistical analyses were performed using STATA-18 (Stata Corporation, College Station, USA).

## Results

Data included a total of 318 female trail runners, with a mean age of 38.3 (± 9.8) years, body mass index of 22.1 (± 2.5) kg/m^2^, years practicing trail running 6.6 (± 5.4) years, training hours per week 7.8 (± 4.9), and participation in 4.9 (± 2.8) competitions per year. A total of 60 (18.9%) participants had a positive PHQ-9 score (defined as a score ≥ 10), screening positive for major depression, and 25 (7.9%) participants answered “yes” to question 9 (Q9) of the PHQ-9, indicating a positivie screening for suicidal ideations.

Table 1 shows the sociodemographic characteristics of runners with positive PHQ-9 scores and positive Q9 responses. There was a statistically significant difference in age and employment status between participants at risk of depression and those not at risk. Indeed, the age group > 40 years old was significantly less likely to score ≥ 10 on the PHQ-9, indicating a lower risk of major depression compared to the younger age groups (*p* = 0.033). Meanwhile, unemployed runners were more likely to screen positive for major depression (*p* = 0.004) and for suicidal thoughts, although the later was not significant (*p* > 0.05).

**Table 1:** Socio-demographic characteristics.

Participants’ characteristics	Sample size	PHQ-9 ≥ 10 (at risk for major depression)		PHQ-9 Q9 = Yes (positive screening for suicidal thoughts)	
	(*N* = 318)	(*N* = 60)	*p* value	(*N* = 25)	*p* value
Age (years)			0.033		0.273
≤30	73 (23.0%)	17 (23.3%)		6 (8.2%)	
31–40	124 (39.0%)	29 (23.4%)		13 (10.5%)	
>40	121 (38.0%)	14 (11.6%)		6 (5.0%)	
Weight (kg)			0.460		0.527
≤55	92 (28.9%)	15 (16.3%)		5 (5.4%)	
56–64	135 (42.5%)	24 (17.8%)		11 (8.2%)	
>64	91 (28.6%)	21 (23.1%)		9 (9.9%)	
Height (cm)			0.593		0.387
≤160	80 (25.2%)	12 (15.0%)		7 (8.8%)	
161–169	154 (48.4%)	31 (20.1%)		9 (5.8%)	
>169	84 (26.4%)	17 (20.2%)		9 (10.7%)	
BMI (kg/m2)			0.178		0.615
≤20	59 (18.6%)	11 (18.6%)		5 (8.5%)	
21–24	196 (61.6%)	31 (15.8%)		13 (6.6%)	
>24	63 (19.8%)	18 (28.6%)		7 (11.1%)	
Nationality			0.423		0.652
Europe	180 (56.6%)	31 (17.3%)		13 (7.2%)	
Non Europe	138 (43.4%)	29 (20.9%)		12 (8.7%)	
Civil status			0.120		0.308
Single	81 (25.5%)	21 (25.9%)		10 (12.4%)	
Married	118 (37.1%)	15 (12.7%)		6 (5.1%)	
Partnership	102 (32.1%)	20 (19.6%)		8 (7.8%)	
Divorced	17 (5.3%)	4 (23.5%)		1 (5.9%)	
Education level			0.334		0.794
High school	30 (9.4%)	8 (26.7%)		2 (6.7%)	
Bachelor	131 (41.2%)	28 (21.4%)		9 (6.9%)	
Master	123 (38.7%)	20 (16.3%)		12 (9.8%)	
Doctorate	34 (10.7%)	4 (11.8%)		2 (5.9%)	
Employment status			0.004		0.075
Employed	229 (72.0%)	41 (17.9%)		15 (6.6%)	
Self-employed	63 (19.8%)	8 (12.7%)		5 (7.9%)	
Unemployed	26 (8.2%)	11 (42.3%)		5 (19.2%)	
Smoker			0.883		0.567
No	267 (84.0%)	50 (18.7%)		22 (8.2%)	
Yes	51 (16.0%)	10 (19.6%)		3 (5.9%)	

Participants running history is presented in Table 2. Performance level, trail running distance, weekly training hours, and yearly race participation showed no significant differences in either the PHQ-9 score or the response to question 9. However, participants who had been trail running fewer than 3 years were more likely to have a positive PHQ-9 score (*p* = 0.002), with no corresponding difference in the response to question 9. Additionally, a statistically significant difference was found for the weekly training distances, showing that participants who ran more than 50 km per week had a higher risk of screening positive for suicidal ideations (*p* = 0.020) but not of major depression.

**Table 2:** Trail running characteristics.

Participants’ characteristics	Sample size	PHQ-9 ≥ 10 (at risk for major depression)		PHQ-9 Q9 = Yes (positive screening for suicidal thoughts)	
	(*N* = 318)	(*N* = 60)	*p* value	(*N* = 25)	*p* value
Total years trail running			0.002		0.677
≤3	115 (36.2%)	32 (27.8%)		10 (8.7%)	
>3	203 (63.8%)	28 (13.8%)		15 (7.4%)	
Performance level			0.504		0.479
Elite	16 (5.0%)	2 (12.5%)		2 (12.5%)	
Non-Elite	302 (95.0%)	58 (19.2%)		23 (7.6%)	
Trail running distance			0.086		0.396
Short trail <42 km	181 (56.9%)	27 (14.9%)		11 (6.1%)	
Long trail 42-80 km	97 (30.5%)	25 (25.8%)		10 (10.3%)	
Ultra trail >80 km	40 (12.6%)	8 (20.0%)		4 (10.0%)	
Weekly training (hours)			0.682		0.070
0–5	108 (34.0%)	18 (16.7%)		4 (3.7%)	
6–9	117 (36.8%)	22 (18.8%)		14 (12.0%)	
≥10	93 (29.2%)	20 (21.5%)		7 (7.5%)	
Weekly training (km)			0.559		0.020
≤50	196 (61.6%)	35 (17.9%)		10 (5.1%)	
>50	122 (38.4%)	25 (20.5%)		15 (12.3%)	
Yearly race participation			0.782		0.413
0–3	124 (39.0%)	22 (17.7%)		7 (5.7%)	
4–6	120 (37.7%)	22 (18.3%)		10 (8.3%)	
≥7	74 (23.3%)	16 (21.6%)		8 (10.8%)	

Table 3 presents the medical and gynecological-obstetrical characteristics. Participants with a history of previous pregnancies had a lower risk of major depression (*p* = 0.013) and suicidal ideation (*p* = 0.008). Similarly, participants with a history of previous births had a lower risk of major depression (*p* = 0.013) and suicidal ideation (*p* = 0.033). No significant differences were found for menopause, the use of oral contraceptives, libido during training, urinary incontinence, pelvic prolapse, or stress fractures. However, a prior diagnosis of an eating disorder was strongly associated with a positive screening for suicidal thoughts (*p* < 0.001) but not the risk of major depression. Runners reporting a decreased libido during training were more likely to screen positive for suicidal thoughts, although this difference did not reach significance (*p* = 0.083).

**Table 3:** Medical and gynecological-obstetrical characteristics.

Participants’ characteristics	Sample size	PHQ-9 ≥ 10 (at risk for major depression)		PHQ-9 Q9 = Yes (positive screening for suicidal thoughts)	
	(*N* = 318)	(*N* = 60)	*p* value	(*N* = 25)	*p* value
Total past pregnancies			0.013	19 (11.8%)	0.008
Never	161 (50.6%)	39 (24.2%)			
≥1	157 (49.4%)	21 (13.4%)		6 (3.8%)	
Total past births			0.013		0.033
Never	177 (55.7%)	42 (23.7%)		19 (10.7%)	
≥1	141 (44.3%)	18 (12.8%)		6 (4.3%)	
Menopause			0.415		0.423
No	276 (86.8%)	54 (19.6%)		23 (8.3%)	
Yes	42 (13.2%)	6 (14.3%)		2 (4.8%)	
Oral contraceptives			0.260		0.711
No	232 (73.0%)	42 (18.1%)		19 (8.2%)	
Yes	44 (13.8%)	12 (27.3%)		4 (9.1%)	
Menopause	42 (13.2%)	6 (14.3%)		2 (4.8%)	
Libido during training			0.343		0.083
It stays the same	164 (51.6%)	26 (15.9%)		8 (4.9%)	
It decreases	105 (33.0%)	24 (22.9%)		13 (12.4%)	
It increases	49 (15.4%)	10 (20.4%)		4 (8.2%)	
History of urinary incontinence			0.939		0.816
No	171 (53.8%)	32 (18.7%)		14 (8.2%)	
Yes	147 (46.2%)	28 (19.1%)		11 (7.5%)	
Urinary incontinence during trail			0.421		0.726
No	176 (55.3%)	36 (20.5%)		13 (7.4%)	
Yes	142 (44.7%)	24 (16.9%)		12 (8.5%)	
History of pelvic prolapse			0.216		0.246
No	303 (95.3%)	59 (19.5%)		25 (8.3%)	
Yes	15 (4.7%)	1 (6.7%)		0 (0.0%)	
History of stress fractures			0.782		0.158
No	272 (85.5%)	52 (19.1%)		19 (7.0%)	
Yes	46 (14.5%)	8 (17.4%)		6 (13.0%)	
Eating disorder diagnosis			0.136		<0.001
No	278 (87.4%)	49 (17.6%)		16 (5.8%)	
Yes	40 (12.6%)	11 (27.5%)		9 (22.5%)	

**Figure 1:**
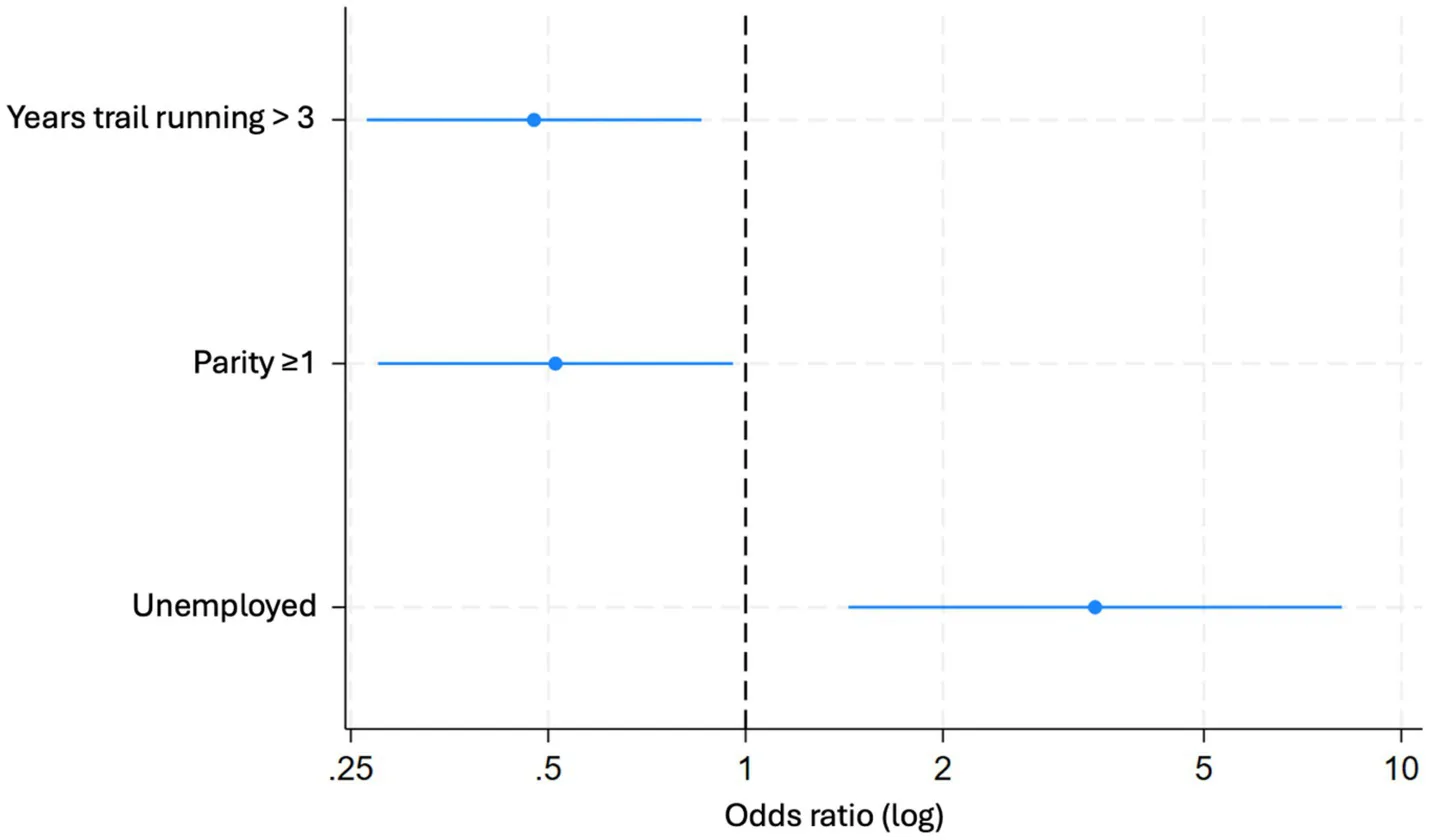
Factors associated with PHQ-9 ≥ 10 (*n* = 318).

With all variables from Tables 1–3 considered, unemployment was the only independent factor positively associated with a PHQ-9 score ≥ 10 (*p* = 0.005, OR 3.41, 95%CI 1.44–8.12), whereas more than 3 years of trail running (*p* = 0.013, OR 0.48, 95%CI 0.26–0.86) and parity ≥ 1 (*p* = 0.036, OR 0.51, 95%CI 0.27–0.96) were inversely associated with a positive PHQ-9 score (Figure 1).

## Discussion

This study aimed to investigate the frequency of depression and suicidal thoughts, and to identify associated factors among female trail runners, addressing a lack of research focusing on women in this sport.

Results showed that 18.9% of female trail runners screened positive for major depression, more than twice the rate in the general female population [World Health Organization (WHO), 2025], but comparable to data in athlete populations (Scheer et al., 2025; Röthlin et al., 2023; Rice et al., 2016). The prevalence of current suicidal thoughts among female trail runners was of 7.9%, which is also nearly twice as high as in the general population (Castillejos et al., 2021). Other studies have also shown a prevalence of 8% among endurance and ultra-endurance runners, male and female (Scheer et al., 2025).

Factors associated with major depression included age, employment status, total years trail running, past pregnancies and past births. Younger female runners reported more depressive symptoms, a finding that has also been observed in a recent study of endurance runners (Scheer et al., 2025). Additionally, unemployment was identified as an independent risk factor for depression, consistent with previous studies demonstrating its significant role in increasing the risk of depression in the general population (Paul and Moser, 2009; Demiral et al., 2022).

Trail running could contribute to the onset or exacerbation of depressive symptoms through factors such as high training loads, challenging environmental conditions, injury, and performance-related pressure. These physical and psychological stressors may negatively impact mood, although our findings do not identify higher training load, elite status or stress fractures to be risk factors for depression. Alternatively, individuals experiencing depressive symptoms may be drawn to trail running as a means of emotional regulation or for the therapeutic benefit derived from exercise and nature exposure. This is supported by our finding that runners who had started trail running more recently were more likely to screen positive for major depression compared to those with a longer history of trail running. These results suggest that women experiencing depressive symptoms may initially turn to trail running as a form of self-medication, which appears to have a beneficial effect over time. However, given the cross-sectional design of the study, the direction of causality cannot be established. Alternatively, women who remain engaged in trail running over many years may represent a more resilient subgroup, as those experiencing mental health difficulties may be more likely to drop out of the sport early. In addition, longer participation in trail running may be associated with factors such as older age or greater socioeconomic stability, which could independently contribute to better mental health outcomes.

Another protective factor for depression as well as suicidal ideation was having given birth at least once, which can be interpreted as having children. This is consistent with a study that found that nulliparous women had higher odds of developing depression (Zhang et al., 2025; Grundström et al., 2024; Kravdal et al., 2017; Radó, 2020; Nelson et al., 2013). Having children provides social support and a greater sense of meaningfulness (Grundström et al., 2024; Nelson et al., 2013), reducing risk of depression and suicidal ideation. Another explanation is that depression is associated with a lower likelihood of having children (Golovina et al., 2023). Alternatively, this association may be influenced by others factors such as age, relationship status, socioeconomic circumstances, or social support, all of which may influence mental health.

Additional factors associated with a positive screening for suicidal ideation included the amount of weekly training kilometers and eating disorder diagnosis. We found that women who practiced trail running more than 50 kilometers per week were more at risk of screening positive for suicidal ideation. This aligns with evidence indicating that the relationship between physical activity and mental-health follows a U-shaped curve. While moderate exercise improves mood, intense exercise can lead to mood deterioration (Thuany et al., 2023; Peluso and Guerra de Andrade, 2005; Shimura et al., 2023; Chekroud et al., 2011; Scheer et al., 2022). In this context, training more than 50 kilometers a week likely represents a level of physical activity that is excessively intense and prolonged, which has been linked to impairment of mental health (Shimura et al., 2023). Moreover, trail running being an individual sport practiced in remote areas, increases the time spent in isolation, which is linked to increased risk of suicidal ideation (Motillon-Toudic et al., 2022). Alternatively, it is possible that women, experiencing suicidal thoughts and that have turned to trail running as an outlet to improve their well-being, tend to run more. It is also possible that these associations are partly explained by lifestyle-related factors. In our sample, 62% of women running more than 50 km per week did not have children, suggesting that high training volume may represent a distinct lifestyle profile. The observed association with a positive screening for suicidal ideation may partly reflect broader differences in life circumstances rather than training volume alone. It is important to note that the PHQ-9 Q9 does not differentiate between thoughts of self-harm and suicidal intent. In the context of endurance sports, the “hurting yourself” component may be interpreted differently, as tolerance of physical discomfort is normalized (Roebuck et al., 2018). Nevertheless, anyone screening positive in this question requires urgent clinical assessment (Rossom et al., 2017). We also found that female trail runners with an eating disorder diagnosis were more likely to screen positive for suicidal thoughts. This is consistent with previous studies reporting that individuals with eating disorders experience elevated rates of suicidal ideation (Amiri and Khan, 2023; Ahn et al., 2019). An eating disorder diagnosis was nearly three times more common among high-volume runners than among those running less than 50 km per week (20% vs. 7%). As excessive exercise can be a component of eating disorders (Campbell et al., 2025), this diagnosis may partially confound the association between high training volume and suicidal ideation, making it difficult to determine the independent contribution of each factor. Finally, we observed a modest association between suicidal thoughts and decreased libido. It has been shown that suicidal thoughts are associated with sexual dysfunction (Sevda et al., 2025). However, energy deficiencies, frequently observed in endurance sports and leading to hormonal changes, can also disrupt sexual function (Logue et al., 2020). Furthermore, a decrease in libido can itself be a source of psychological distress, loss of self-esteem, and isolation, thereby reinforcing suicidal thoughts.

These findings highlight the need for structured mental health support within the trail running community. Identifying factors associated with an increased risk of depression is essential for developing targeted prevention strategies and improving the early identification of athletes who may be more vulnerable. To effectively address mental health in trail running, it may be useful to promote conversations about mental health to reduce stigma (Cosh et al., 2024), incorporate mental skills training and management techniques for athletes (Purcell et al., 2019), and provide access to professional support like sports medicine specialists and psychologists (Reardon et al., 2019). Educational workshops that raise awareness about common mental health challenges could be integrated into training programs. These workshops, designed for coaches and support staff, significantly increase participants’ knowledge of the signs and symptoms of depression as well as their confidence to support someone who may be struggling with mental health disorders (Sebbens et al., 2016; Bapat et al., 2009; Kitchener and Jorm, 2002; Hadlaczky et al., 2014). Incorporating these approaches would help raise awareness within the trail running community.

A limitation of the present study is that it relied on anonymous self-reported data and therefore the responses or clinical diagnoses could not be confirmed. The PHQ-9 is a widely validated and recognized screening tool for depression. However, a definitive diagnosis requires verifying responses in the context of additional clinical information (Costantini et al., 2021). A further limitation is the use of a single item from the PHQ-9 as a substitute for suicidal thoughts. This approach is commonly used in research, as there is currently no definitive screening method for accurately determining suicide risk (Andreotti et al., 2020). However, accurate assessment requires a combination of self-report measures, direct questioning and clinical evaluation conducted by health-care professionals. Future studies should incorporate validated multi-method qualitative assessments, although, such approaches may reduce feasibility in large populations. Additionally, only a limited set of potentially associated factors was examined. Other variables previously linked to depression and suicidal thoughts in the general population, such as family history, social support, or adverse life events [World Health Organization (WHO), 2025; Favril et al., 2022; Ivey-Stephenson et al., 2022], were not assessed in this cohort. Finally, our study had a small number of cases reporting suicidal ideation, which may have limited the statistical power to detect significant associations.

To our knowledge, this is the first study to investigate depression and suicidal thoughts specifically among female trail runners. Because mental health outcomes result from numerous interacting factors across an individual’s life, trail running itself may not be causal, rather that individuals with pre-existing mental health challenges may be drawn to the sport as a form of self-medication. However, the cross-sectional design of our study does not allow us to determine the direction of causality. Longitudinal studies are required to clarify underlying mechanisms and better understand this association, and findings from this study should not be generalized to all trail running populations.

## Data Availability

The raw data supporting the conclusions of this article will be made available by the authors, without undue reservation.
